# Autochthonous *Trypanosoma* spp. in European Mammals: A Brief Journey amongst the Neglected Trypanosomes

**DOI:** 10.3390/pathogens10030334

**Published:** 2021-03-13

**Authors:** Alice Magri, Roberta Galuppi, Marialetizia Fioravanti

**Affiliations:** Department of Veterinary Medical Sciences, Alma Mater Studiorum-University of Bologna, Ozzano Emilia, 40064 Bologna, Italy; alice.magri3@unibo.it (A.M.); marialeti.fioravanti@unibo.it (M.F.)

**Keywords:** *Trypanosoma* spp., mammals, Europe, epidemiology, *T. theileri*, *T. lewisi*, *T. grosi*

## Abstract

The genus *Trypanosoma* includes flagellated protozoa belonging to the family Trypanosomatidae (Euglenozoa, Kinetoplastida) that can infect humans and several animal species. The most studied species are those causing severe human pathology, such as Chagas disease in South and Central America, and the human African trypanosomiasis (HAT), or infections highly affecting animal health, such as nagana in Africa and surra with a wider geographical distribution. The presence of these *Trypanosoma* species in Europe has been thus far linked only to travel/immigration history of the human patients or introduction of infected animals. On the contrary, little is known about the epidemiological status of trypanosomes endemically infecting mammals in Europe, such as *Trypanosoma* *theileri* in ruminants and *Trypanosoma* *lewisi* in rodents and other sporadically reported species. This brief review provides an updated collection of scientific data on the presence of autochthonous *Trypanosoma* spp. in mammals on the European territory, in order to support epidemiological and diagnostic studies on Trypanosomatid parasites.

## 1. Introduction

The genus *Trypanosoma* includes flagellated protozoans belonging to the Trypanosomatidae family (Euglenozoa, Kinetoplastea) that can infect humans and several animal species [1]. They are mostly dixenous parasites, meaning that the presence of two hosts (commonly one vertebrate and one invertebrate) is required in order to complete their life cycle. Such organisms are capable of parasitizing a wide range of vertebrate hosts, from mammals to birds, fish, amphibians, and reptiles [2].

The most studied species are those causing serious diseases in humans, and are not endemic in the European continent. This group includes species of the *Trypanosoma brucei* complex, mainly responsible for African trypanosomiasis [3], which are usually transmitted cyclically through a salivarian route by the tsetse fly (*Glossina* spp.), or rarely by congenital transmission [4]. In particular, the subspecies *T. brucei gambiense* and *T. brucei rhodesiense* are responsible for human African trypanosomiasis (HAT), also known as sleeping sickness, which can result in death of the patient if untreated [5,6]. A variety of wild and domestic animal species may act as reservoir in endemic countries, especially for *T. brucei rhodesiense* [7,8]. In Europe, the diagnosis of HAT is usually related to travel or migration [9,10,11,12,13,14,15,16,17,18], with some differences concerning the species isolated; in general, rhodesiense HAT is more connected with tourism, particularly with travelers returning from short visits to endemic countries, and is the most frequently diagnosed, while gambiense HAT patients had been living in endemic countries for extended period, and therefore is more related to history of migration with economic connections to the endemic countries [18,19].

Moreover, *T. cruzi* is responsible for human American trypanosomiasis, the Chagas disease, typically acquired through stercorarian transmission by triatomine bugs (reduviid insects) vector species, although vertical and iatrogenic transmission are also described [20]. The Chagas disease is endemic in Central and South America, where it has also been described in more than 100 animal species, whose role as reservoir is well established [21,22,23]. A large number of cases have also been reported in Europe, both in travelers and, in particular, in migrants from endemic countries; this phenomenon has increased particularly since the 1990s due to massive migrations from Latin America to Italy, Portugal, and Spain [20,24,25,26,27], as well as to other European countries such as Belgium, France, Germany, the Netherlands, Switzerland, and the United Kingdom [28].

In domestic animals, animal African trypanosomiasis (AAT, also named nagana) is described as an acute or chronic disease caused by several species of *Trypanosoma* including *T. brucei* subsp. *brucei*, *T. vivax*, *T. congolense*, *T. simiae*, and *T. suis* [7]. These trypanosomes are cyclically transmitted by tsetse fly, although *T. congolense* and *T. vivax* might be mechanically transmitted by Tabanids and Stomoxines [29]. Although evidence for the epidemiological relevance of theirmechanical transmission in Africa are scant, such route has allowed these species to expand their range beyond that of *Glossina* spp. In particular, *T. vivax* has expanded its distribution to South and parts of Central America during European colonization in the last centuries [30]. In Europe, autochthonous cases of nagana have not been described thus far [31,32]. Clinical manifestations may vary according to the species; in particular, *T. congolense* present in Sub-Saharan Africa causes large economical losses in endemic countries [3].

Concerning other animal trypanosomes, such as *T. brucei evansi* and *T. brucei equiperdum*, the possibility of spreading in Europe is different. Classification of these species (previously named as *T. evansi* and *T. equiperdum* [2]) is still subject of debate; since they share important morphological and genetic traits, both parasites should be considered subspecies of *T. brucei* [33,34,35]. *T. brucei evansi* is the causative agent of the animal disease surra, which can affect a wide range of mammals from different geographical areas—camels, horses, buffalos, and cattle are particularly affected, although other animals, including wildlife, are also susceptible. Being transmitted in a non-cyclic way by tabanids, other flies, vampire bats, or carnivores, surra’s spatial distribution is wide, including Africa, Asia, and Latin America [36]. *T. brucei evansi* has been known to be present since 1997 in the Canary Islands [37,38], where the most important population of dromedary camel (*Camelus dromedarius*) in Europe is present [39], and *Stomoxys calcitrans* is commonly involved in its transmission in the archipelago [40]. In 2010, following control programs, in the island of Gran Canaria, about 5% of the camelid population remained positive [40], and it was supposed that small ruminants, rodents, or rabbits could play a role as reservoirs of infection, although no evidence of the parasite in rodents was found [41]. Surra outbreaks have also been reported in dromedary camels and equids (horses and donkeys) from mainland Spain and France following importation of camelids from Canary Island [39,42]; nevertheless, in these cases, sanitary measures were successful in controlling the disease [40]. Furthermore, a single case of Surra was described in Germany in a Jack Russel dog imported from Thailand [43]. Further outbreaks in continental Europe have not been reported. Surveillance measures should be considered by European Countries for current risk of introduction; however, according to the European Food Safety Authority (EFSA), it is currently inconclusive whether *T. brucei evansi* infections (including surra) can be considered eligible to be listed for Union intervention in Animal Health Law [44].

*T. brucei equiperdum*, the causative agent of dourine in equids, represents an exception amongst trypanosomes, being the only species transmitted directly between hosts through coitus [45]. Dourine was anciently described in North Africa, but the etiological agent was first isolated only at the beginning of the last century by Buffard and Schneider [46]. In Europe, the disease has been described from the XVIII century in Russia, as well as in France, due to introduction of Persian, and Syrian and Spanish stallions, respectively [2]. After the Second World War, the disease spread in Europe, but thanks to several control efforts aimed at eradicating dourine, the disease disappeared from western and central European countries [47]. Although sporadic outbreaks were reported in the 1970s in Italy, dourine remained unreported until 2011, when five outbreaks were confirmed, once again in Italy [48,49]. Such disease is still considered a relevant health issue for equines and represents a trade barrier in the movement of horses [50]; since it needs no vector for its transmission and can spread with the host, it requires implementation of official control plans [51].

**Figure 1:**
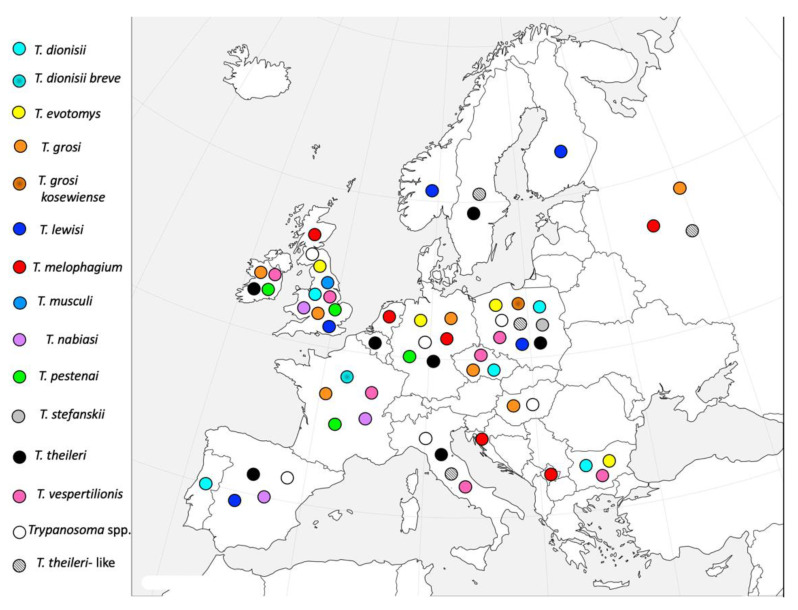
Distribution of the autochthonous *Trypanosoma* species in European mammals.

Along with these well-known and studied species, other *Trypanosoma* spp. can infect mammals, and some of them are also diffused in Europe. The aim of this brief review was to gather reports of findings of these neglected species in Europe in order to raise awareness on the presence if these flagellates during epidemiological and diagnostic studies on trypanosomatid parasites on the European territory (Figure 1).

## 2. General Taxonomy of the Genus *Trypanosoma*

In order to properly define the distribution of *Trypanosoma* spp. in Europe, we deemed a brief section concerning the classification of this genus to be opportune.

As previously mentioned, trypanosomes are obligate parasites belonging to the Protozoa subkingdom, phylum Euglenozoa, class Kinetoplastea, order Trypanosomatida [1,52]. Kinetoplastea are characterized by the presence of a modified mitochondrion containing a body constituted of a disc-shaped, DNA-containing organelle, known as kinetoplast (from which the class name is derived), located beside the kinetosome at the base of the flagellum [53]. The classification of the Trypanosomatidae family is extremely complicated and still subject of debate amongst parasitologists for several reasons, among which the lack of morphological differences between phylogenetically distinct taxa and of an unequivocal classification approach [1,54]. 

The genus *Trypanosoma* is usually classified in the Blechomonadinae subfamily, which predominantly hosts dixenous parasites [55] and is conventionally divided in two groups on the basis of the replication site inside the invertebrate host “Salivaria”, which develops in the foregut and is transmitted by inoculation, and “Stercoraria”, which develops in the hindgut and therefore is transmitted by fecal contamination of skin injuries or mucosae [56]. Although this classification is not strictly taxonomic, it is still widely used because easily recalls life cycle and infection route of trypanosomes and will be also utilized in this review. In mammalian host, the salivarian trypanosomes reproduce in the trypomastigote stage that has the kinetoplast in terminal or subterminal position and blunt posterior end. The Salivaria group includes four subgenera: *Duttonella*, *Nannomonas*, *Pycnomonas*, and *Trypanozoon*, mainly transmitted by tsetse flies [3,57]. Slight morphological differences between the subgenera have been described: *Duttonella* has rounded posterior end with large and terminal kinetoplast, *Nannomonas* has the kinetoplast in marginal position, while *Pycnomonas* has small and subterminal kinetoplast [58]. Assuming the progressive adaptation of trypanosomes to the tsetse fly as indicative of evolution, researchers have considered the subgenus *Duttonella* (non-cyclic) as the most ancient, and *Trypanozoon* the most recent [56,59,60]. The *Duttonella* subgenus includes *T. vivax*, responsible for nagana diseases in various animal species in Africa, or asymptomatic infections in Central and South America [61]. Concerning the subgenus *Nannomonas*, it includes species of interest in animal health such as *Trypanosoma simiae*, *Trypanosoma godfreyi*, and *Trypanosoma congolense*, also causing nagana in animals [62]. *Pycnomonas* subgenus includes *T. suis*, causing nagana in Suidae in Africa [63]. The *Trypanozoon* subgenus comprises cyclically transmitted trypanosomes extremely relevant for human and animal health, such as *T. brucei* complex, including *T. brucei brucei*, also an agent of nagana disease in animals in Africa, and the HAT causal agents *T. brucei rhodesiense* and *T. brucei gambiense* [64]. Moreover, this subgenus includes *T. brucei evansi*, which causes Surra in a wide range of hosts, and the monoxenous sexually transmitted *T. brucei equiperdum* [36,65].

The Stercoraria group comprises protozoans that, in the mammalian host, reproduce as epimastigote/amastigote forms, and present not reproducing trypomastigote forms in blood. The latter ones have a large kinetoplast, usually not terminal, and pointed end of the body. Stercoraria are mostly considered as non-pathogenic (except for *T. cruzi*) and comprise different subgenera [66]: (i) *Schizotrypanum*, with trypomastigotes typically curved with kinetoplast close to the posterior end of the body, includes *T. cruzi* responsible for Chagas disease or human American trypanosomiasis [67,68]; (ii) *Megatrypanum* are large trypanosomes that in trypomastigote forms have kinetoplast near the nucleolus, far from the posterior end [58] and include, amongst others, the worldwide distributed cyclic species *Trypanosoma melophagium* in sheep and *Trypanosoma theileri* in cattle [2]; (iii) *Herpetomonas,* defined as subgenus by Molyneux [69], includes *Trypanosoma lewisi* as the most studied species, long isolated in rodents worldwide and lately occasionally reported also in humans in Asia and Africa [70,71]. The trypomatigote forms are medium-sized with slender curved body and pronounced free flagellum [2].

According to data based on molecular sequences retrieved from GenBank (non-taxonomic), we found that not all trypanosomes can be classified according to these subgenera; therefore, in this classification, two clades have been introduced: firstly, the clade of “*Trypanosoma* with unspecified subgenus”, in which some parasites of wild fauna, such as *Trypanosoma evotomys, Trypanosoma grosi*, *Trypanosoma nabiasi*, and *Trypanosoma pestanai,* are included. Such parasites are commonly defined as non-pathogenic, as they have rarely been isolated in course of clinical disease. Unexpectedly, according to phylogenetic analysis, *T. theileri* belongs to this clade, although it is taxonomically included in the *Megatrypanum* subgenus. The second clade with no specific subgenus is referred to as “unclassified trypanosomes”, further subdivided into “fish trypanosomes” and “other trypanosomes”; the latter includes recently discovered trypanosomes waiting for proper classification. Table 1 reports a schematic classification of *Trypanosoma* spp. on the basis of data retrieved from GenBank taxonomy [72].

## 3. *Trypanosoma* Species Naturally Occurring in Domestic and Wild Mammals in Europe

As seen in the literature, a wide variety of *Trypanosoma* species have been reported to infect mammals from all European Countries (Table 2). Such species are commonly reported as non-pathogenic trypanosomes. In general, certain characteristics usually distinguish non-pathogenic trypanosome species from the pathogenic ones: (i) the host–parasite relationships are well adapted evolutionarily to both vertebrate and invertebrate host; (ii) infection rates in vectors are high; (iii) infected mammals are usually healthy carriers with inapparent, nonchronic infections; (iv) the host range is extremely restricted; (v) in an invertebrate host, the development of metacyclic trypomastigote occurs in the hindgut and the parasites are shed with the feces; (vi) in the mammalian host, trypanosomes shortly reproduce as epimastigote and/or amastigote forms, after which non-reproductive trypomastigote forms circulate in blood [66]. The only exception to this classification scheme is *T. cruzi*, which is not naturally present in Europe and, although included in the Stercoraria group, is highly pathogenic.

### 3.1. Trypanosoma theileri

While drafting this review, the presence of *T. theileri* Laveran, 1902 has shown great relevance in Europe. As previously mentioned, this species is usually classified in the *Megatrypanum* subgenus, however, in terms of phylogenetic analysis, it is grouped within the “*Trypanosoma* with unspecified subgenus”. *T. theileri* is considered a mildly pathogenic species that typically infects wild and domestic ruminants [131]. Different tabanid species are common vectors of *T. theileri*, transmitting the pathogen by laying infected feces on the skin of the mammalian host or by ingestion of infected insects [127]; however, during a study concerning *Leishmania infantum* in the Emilia-Romagna region (Italy), the presence of *T. theileri*-like trypanosomes has been recently reported in sandflies (*Phlebotomus* spp.) [149], although their role as vectors has not been established. Exploiting abraded skin or mucosae, *T. theileri* invades the bloodstream of the mammalian host; prepatent period ranges from 4 to 20 days and parasitemia decreases after 2–4 weeks [66]. First isolation was performed in Africa by the veterinary bacteriologist Arnold Theiler, who observed animals with clinical manifestations similar to the “Gall-sickness” (currently Anaplasmosis) during immunization of cattle against rinderpest. He observed *Trypanosoma*-like forms in blood smears and sent them to French and British researchers (Laveran and Bruce, respectively), who both named the parasite as *T. theileri*; however, since Laveran published the description earlier, the species was credited to him [2,160,161]. After the first isolation, reports have been numerous but often incorrect due to the great morphological variability of strains isolated from different animal hosts and geographical areas. In fact, several authors named different new species (e.g., *Trypanosoma frank* from cattle in Germany, *Trypanosoma wrublewskii* from the European bison *Bison bonasu* in Poland, *Trypanosoma americanum* and *Trypanosoma rutherfordi* from cattle in North America [2]), which were lately recognized as *T. theileri* by Herbert [162], making it clear that its distribution was wider than the African continent. Until 1970, reports of *T. theileri* in cattle ranged from Australia [163], to the United Kingdom [158,159], to the USA and Canada [164,165]. Concerning Europe, this species has more recently been reported from cattle also in Belgium, Germany, Italy, Ireland, Poland, and Spain [123,124,128,130,131,137]. Although these infections are generally reported as asymptomatic, clinical manifestations have sometimes been described, as primarily referred by Theiler [161]. Cases of illness were reported mostly in immunocompromised animals consisting of mild leukocytosis, enlargement of the spleen, anemia, weight loss, and considerable drop in milk production, especially if the infection concurs with bovine leukemia virus [128,129,130,131]. Water buffalo (*Bubalus bubalis*) is also susceptible to infection, and recent casual findings have been described in Italy in both cattle and water buffalo [128,129], whereas, in Poland, reports in European bison are numerous [132,133,134,135].

In Europe, *Megatrypanum* species, often morphologically described as *T. theileri*-like, were also reported in wild ruminants such as roe deer (*Capreolus capreolus*), fallow deer (*Dama dama*), and red deer (*Cervus elaphus*) [114,148,150,151,152,153,154,166]. *T. theileri*-like strains were also detected by molecular biology in vectors (i.e., tabanid flies) in Russia [149] and Poland [147]. Studies concerning the characterization of *Megatrypanum* trypanosomes from European Cervidae using isoenzyme analysis and pulsed-field gel electrophoresis suggested that there should be at least two *Megatrypanum* species infecting European deer, one in roe deer, and one in fallow and red deer, and differing from *T. theileri* affecting cattle [127]. More recent studies on phylogenetic analysis of *Megatrypanum* trypanosome from cattle, water buffalo, deer, and antelopes revealed the presence of several host-specific genotypes [167]. Unfortunately, the assessment of pathogenic effects and clinical course of *Trypanosoma* spp. infections in the wild fauna is generally challenging and the diagnosis rely mostly on post-mortem examination, which only in few cases has allowed to detect poor general conditions and small size in infected animals [148,168].

The presence of trypanosomes in roe deer was also reported in Poland by Kingston et al. [122] during one of the few epidemiological studies focused on such parasites in wild fauna in Europe. Blood from hunted roe deer killed between August 1984 and July 1988 revealed a prevalence of 66.6%. The *Trypanosoma* found differed from any others isolated from wild ruminants in central Europe and North America, and a consistent percentage of protozoa lacked a free flagellum, assumed by the authors to be the vector-infective form. Therefore, a new species in the subgenus *Megatrypanum* was described on the basis of morphological traits, namely, *Trypanosoma stefanskii*. No sequence was deposited in GenBank and, to our knowledge, no other reports of this species occur.

On a diagnostic perspective, *T. theileri* and other *Megatrypanum* trypanosomes of ruminants often represent an occasional finding occurring in other investigations [128,149]. For example, Galuppi et al., during cattle blood culture trials for the cultivation of piroplasms [169], observed the presence of *Trypanosoma* sp. (R.G., personal communication). As it emerges, reports of these trypanosomes are often occasional and the actual prevalence in domestic and wild fauna is not known.

### 3.2. Trypanosoma melophagium

Amongst *Megatrypanum* trypanosomes, *T. melophagium* is species-specific for domestic sheep and is transmitted by the sheep ked *Melophagus ovinus*. Ked become infected through the blood of parasitized animals and, after multiplication in the digestive system of the fly, the metacyclic form develops in the hindgut. Sheep acquire the infection by eating infected ked [2,163]. The first report of *T. melophagium* was from Germany, where in 1905 Pfeiffer observed the presence of “trypanosome-like flagellates” in sheep ked [98]. At first, studies failed in proving the presence of the protozoan in sheep blood and *T*. *melophagium* was classified as a parasite of the ked gut and named *Crithidia melophagia* [170,171]. Few years later Woodcock succeeded in observing trypanosomes in fresh sheep’s blood and identified them as developmental stages of the flagellates described in sheep ked [103]. Interestingly, his work was hardly criticized, and only after almost 10 years of controversy, Nöller [172] and Kleine [173], in separate studies proved not only that *T. melophagium* and *C. melophagia* were actually the same species but also that ked became infected only after feeding on infected sheep. This was finally confirmed by Hoare [104] who, in the same years, found *T. melophagium* in the 80% of the sheep examined in England. Gibson and colleagues observed close genetic similarity between *T. melophagium* and *T. theileri*, suggesting that *T. melophagium* represents a lineage of *T. theileri* that adapted to be transmitted by sheep ked [107]. In more recent years, this protozoan has been eradicated in the United Kingdom as a consequence of the widespread use of pesticides effective against ked [105], with persistency in the Outer Hebrides off the northwest coast of Scotland [106]. Infection with *T. melophagium* is not associated with clinical manifestations, the parasitemia is transitory (3 months), and there is no lasting immunity, and thus sheep can be readily re-infected after several months [104]. Due to the lack of clinical manifestation associated with the infection in sheep, scattered and sporadic reports occurred in Europe, particularly from Germany [100], southeastern Russia [102], former Yugoslavia (currently Croatia and Kosovo) [108], and Turkey [174]. Such sporadic reports could be related to difficulties in the detection of *T. melophagium* in sheep blood samples, possibly due to the low and transitory parasitemia. In fact, a more recent study conducted in Croatia observed a re-emergence of sheep ked in organic farms—the ked were heavily infected by *T. melophagium* (86% of samples), however, none of the 134 sheep from which they had been collected resulted positive at blood smear examination [97].

### 3.3. Trypanosoma lewisi

*T. lewisi* is a cosmopolitan *Herpetosoma* species, also widely distributed in Europe, responsible for infections in rodents, more specifically in rats. *T. lewisi* is perhaps the best studied non-pathogenic trypanosome amongst the ones here presented, probably for its presence in rats used as laboratory animals [2]. Its first observation dates back to 1850 in France, by Chaussat, who referred its finding as nematode larvae in blood [175]; only almost 30 years later was the parasite recognized as a trypanosome species [176]. Dynamics of infection have been largely studied by Minchin and Thompson, who in 1915 published an extremely detailed work on the development of *T. lewisi* in its vector, the rat flea *Ceratophyllus fasciatus* [177].

In rats, the infection occurs without clinical manifestations, and is primarily characterized by the presence of epimastigote forms in peripheral blood. Rat fleas, while feeding upon the rodent’s blood, eliminate feces containing final metacyclic stages, the metatrypanosomes. Through injured skin or mucosae, parasites gain entrance to the host blood stream and multiply as epimastigotes. Several days are needed to detect the parasites in blood and the prepatent period varies according to the parasite load [66]. Recent in vitro studies have also demonstrated the presence of further stages of development and multiplication, such as the “rosette” stage (multiple divisions forms) and the trypomastigote [178].

Although *T. lewisi* is considered cosmopolitan, only few findings have been reported in European wild/synantropic murine population. For instance, it was described in 1970 in Southern Finland, mostly in Helsinki, wherein 36.2% of rats tested positive [94]. Moreover, it was reported in Norway in voles (*Clethrionomys glareolus*, *Microtus agrestis*, and *Apodemus sylvaticus*) [95] and again more recently in voles in Poland [96], but with no prevalence data due to the different aim of the studies (morphological characterization). Rodríguez et al. [41] reported *T. lewisi* in 13% of the rat population examined in the Canary Islands (Spain).

*T. lewisi* can be transmitted to humans, but only few cases have been described, mostly in children from in Asia and Africa, showing a fatal course if untreated [70,71,179]. Recent studies have demonstrated that *T. lewisi* is resistant to trypanolysis operated by human serum, exhibiting characteristics similar to human pathogenic trypanosomes; therefore, its role as human pathogen might be underestimated [180]. In Europe, no human cases due of *T. lewisi* infection have been described thus far.

### 3.4. Trypanosoma nabiasi

*T. nabiasi* was firstly observed in France, where it was reported as an unidentified trypanosome in the blood of wild and domestic rabbits by Jolyet and Nabias [109]. It was lately named as *T. nabiasi* by Raillet in 1895 and considered as a *T*. *lewisi*-like form in the subgenus *Herpetosoma* [2]. It is now phylogenetically included in the “trypanosomes with unspecified subgenus” clade [72]. The life cycle of *T. nabiasi* comprises the flea *Spilopsyllus cuniculi* as a vector [181]. Rabbits become infected after ingestion of the flea or by contamination of injured skin or mucosae with flea feces. Prepatence may vary from 5 to 12 days, while infection lasts from 4 up to 8 months, during which the parasite can be found in the rabbit blood and is infective for fleas; immunity prevents from reinfection [112,182]. *T. nabiasi* was described in wild rabbits (*Oryctolagus cuniculus*) in Great Britain [112] and in Cottontail rabbits (*Sylvilagus* spp.) in North America [183]. More recently, *T. nabiasi* has been reported from rabbits in Spain in coinfection with *Leishmania infantum*, highlighting possible problems occurring in the diagnosis of leishmaniasis in case of co-presence of these flagellates [110,111].

### 3.5. Trypanosomes of Small Rodents

Another *T. lewisi*-like protozoan is *T. evotomys*, a parasite of Arvicolinae rodents, referred to the subgenus *Herpetosoma* [2] and now phylogenetically included in the “trypanosomes with unspecified subgenus” clade [72]. Described for the first time by Watson and Hadwen in 1912 in the Canadian vole (*Evotomys saturates*, now *Clethrionomys glareolus*) [183], it was subsequently found in the United Kingdom [69,80,81,82,83], Germany [78,79], Poland [84,85], and Bulgaria [77]. The vector is yet to be identified, although fleas are possibly involved. In experimental infection via inoculation, prepatence lasts from 5 to 6 days, during which parasites invade and multiply in lymph nodes and spleen. Patent infection typically lasts 1 month or more in splenectomized hosts [77].

*Trypanosoma. grosi* is included within the *T. lewisi*-like group. It was firstly described as “very motile vermicules” by Gros [91] in Russian wood mouse (*A. sylvaticus*) and its recognition as *Trypanosoma* sp. occurred several years later in France by Laveran and Pettit [87]. In Russia, it was at first misidentified (*Trypanosoma apodemi* and *Trypanosoma korssaki*) when found in different vole species [84], and then more recently recognized as synonyms of *T grosi* [2]. Since then, this species has been reported in the United Kingdom [80,82,90], Ireland [90], Germany [88], Hungary [89], and the Czech Republic [86]. Moreover, *T. grosi kosewiense* has been described as a new subspecies of *T. grosi* in Poland from voles (*Microtus* spp. and *Apodemus* spp.) and from the yellow-necked mouse (*Apodemus flavicollis*) [93].

*T. musculi* is a trypanosome of the house mouse (*Mus musculus*), which was first observed in mouse blood in Gambia by Dutton and Todd in 1903 and was defined as a new species of the subgenus *Herpetosoma* by Thiroux in 1905 [184]. It has not been included in any phylogenetic clade yet. Mice acquire the infection from fleas of the genera *Ctenophthalmus*, *Leptopsylla*, and *Nosopsylla* [185]. As happened with *T. lewisi*, the role of *T. musculi* as a potential human pathogen has been questioned, mainly due to the biological and morphological characteristics shared between them. *T. musculi* revealed in vivo and in vitro lower sensitivity to human sera than *T. brucei brucei* but higher when compared to *T. lewisi*. Although no case of trypanosomiasis attributed to *T. musculi* has been reported yet, and infection in healthy humans is considered unlikely, some authors suggest caution, especially in immunocompromised patients [186]. In Europe, the only report available in the literature is from a review on protozoans of British small rodents where *T. musculi* is cited as a parasite of *Mus musculus*; no further data are available [82].

Moreover, during a parasitological study conducted on common shrew (*Sorex araneus*) in Northwest England, 9 out of 76 specimens tested positive for *Trypanosoma* spp. It is not possible to identify parasites at the species level on the basis of molecular data available in GenBank. The strain shared great similarity with *T. lewisi*, but formed an outgroup when clustered with *Trypanosoma microti*, *T. evotomys*, *T. musculi*, *T. lewisi*, and *T. grosi* [157].

### 3.6. Trypanosomes of Bats

Over 30 species of trypanosomes of the *Schizotrypanum* subgenus have been reported in more than 100 Chiroptera species all over the world, including the well-defined *Trypanosoma cruzi*, *T. vespertilionis*, *T. rangeli*, and the globally distributed *T. dionisii* [187]. Trypanosomes in bats were first described in *Miniopterus schreibersii* from Italy in 1899 by Dionisi, who identified it as *T. vespertilionis*; this species was further reported in Italy [140,141], Ireland [142], France [138], and the United Kingdom [143]. In Portugal, Bettencourt and Franca [74] described the species *T. dionisii,* which was later considered as a synonym of *T. vespertilionis*, later reported during specific surveys on trypanosomes in bats in the United Kingdom [144,145]. Only in 1975 did a comparative study based on laboratory culture prove that although these parasites were closely related, they actually differed from a morphological, physiological, and antigenical standpoint [188]. Such species are considered *T. cruzi*-like due to morphological similarities with *T. cruzi* [2]. In the United Kingdom, a later survey conducted on British bats reported the presence of both *T. dionisii* and *T. vespertilionis* [146]. A subspecies of *T. dionisii*, *T. dionisii breve*, was described in France in 1979, and was differentiated on the basis of morphological differences and enzyme electrophoresis [76]; however, to our knowledge, no further reports succeeded.

In bats, trypanosome development follows the *T. cruzi* pattern—infection of the host occurs through injured skin with epimastigote forms invading bloodstream to reach the target organs, namely, striated muscle, cardiac muscle, and stomach muscle (depending on strains/species involved), where parasites multiply as amastigote forms and may form pseudocysts in which trypanosomes multiply by binary fission as epimastigote; rupture of this pseudocysts allows the trypanosome to invade the bloodstream as trypomastigote forms [2]. Vectors of bat trypanosomes are *Cimex* spp. and bat flies (*Nycterida schmidlii*) [75,152]. No report of clinical manifestation has thus far been notified. In a recent study, 381 bat specimens collected in eastern and central Europe between 2015 and 2019 were screened with nested PCR for trypanosomes presence—a part of these tested positive for *T. dionisii* (32.3% in the Czech Republic, 8.3% in Bulgaria, and 16.2% in Poland), while a smaller fraction tested positive for *T. vespertilionis* (3.8% in the Czech Republic). Hematological parameters showed no significant differences between infected and non-infected specimens [73]. In the same year, a survey about the presence of *Trypanosoma* spp. in bats (9 subjects from Hungary, 16 from Italy, and 10 from Spain) and the flies *Nycteribia schmidlii scotti* (71 subjects) parasitizing them, found that in Hungary the prevalence of infection was 33.3% in bats and 35.3% in bat flies, while in Italy it was 43.8% and 11.6%, and in Spain it was 30% and 81.8%, respectively; no species identification was performed [152].

### 3.7. Trypanosoma pestenai

Amongst the trypanosomes naturally infecting wild carnivores, *T. pestanai* is the only one described in the European territory. *T. pestanai* recognizes the badger (*Meles meles*) as preferential host and was first reported in Portugal in 1905 [189]. It was then described in France [113], the United Kingdom [117,118,120,121], and Ireland [116]. In particular, in the United Kingdom, the role of the badger flea (*Paraceras melis*) in the transmission of *T. pestanai* has been recognized [119]. As observed for most of non-pathogenic trypanosomes, the infection in badgers seems to be silent, and not associated with alterations of complete blood count [116]. This parasite has been recently reported in Germany in a 12-year-old beagle with a history of occasional travels to Switzerland, and parasitological investigations based on PCR and blood cell cultivation revealed the concurrent presence of *Anaplasma phagocytophilum* [115].

### 3.8. Trypanosoma *spp.*

Besides reports concerning the aforementioned *Trypanosoma* species, in some cases identification has been carried out only at the genus level [151,155,156]. For example, Olmeda et al. [155] described the presence of protozoans morphologically referable to the *Megatrypanum* subgenus in blood smear from 17 deer shot in Spain; however, no further data are available. Moreover, in Spain, during a study focused on non-lethal parasites of wild rabbit in a re-stocking program, 125 rabbits of different age classes were examined to test the presence of *Trypanosoma* spp. in blood smear, finding a prevalence of 9.5% in young, 4.4% in juveniles, and 8.2% in adults; no morphological or molecular identification was performed [156]. Such findings suggest that further studies are necessary to increase the knowledge on the *Trypanosoma* species circulating in European mammals.

## 4. Closing Remarks

The genus *Trypanosoma* includes a wide number of worldwide distributed species that can affect human and animal health. The most important and studied pathogenic species are responsible for African and South American trypanosomiases. As described in this review, the presence of such species in Europe is typically linked to human or animal immigration/travel/introduction from endemic countries. Such reports do not seem to constitute a threat for the European population due to the life cycle and transmission route strictly depending on vectors that are not present in Europe. On the contrary, occasional findings of *T. evansi* might represent a concern, due to the possible spillover to native hosts, favored by the wide range of vectors involved also in a non-cyclic transmission route.

The presence of autochthonous *Trypanosoma* species described in this review, all referable to non-pathogenic stercorarian trypanosomes, has been documented in Europe since the XIX century in both domestic and wild animals. Due to their scant pathogenic effects on the host, these species are more frequently reported as occasional findings during parasitological surveys not specifically focused on trypanosomes and/or during the molecular diagnosis of other strictly related pathogens, such as *Leishmania* spp. [110,111,146].

Although accidental, such findings are far from being trivial, as they provide useful information on the current epidemiological distribution of trypanosomatids in different geographical areas and hosts [148], with relevant implications also for the improvement of diagnostics [110,111]. Studies aimed to improve our knowledge on their epidemiology in Europe should be encouraged, especially considering that environmental changes could increase their spatial distribution.

## Figures and Tables

**Table 1:** Classification scheme of the *Trypanosoma* species as retrieved from GenBank taxonomy (25 January 2021), modified by referring them also to Salivaria and Stercoraria groups.

Group	Subgenus/Clade	Species	Subspecies
Salivaria	*Duttonella*	*T. vivax*	
	*Nannomonas*	*T. congolese*	
		*T. godfreyi*	
		*T. simiae*	
	*Pycnomonas*	*T. suis*	
	*Trypanozoon*	*T. brucei*	*T. brucei brucei*
			*T. brucei gambiense*
			*T. brucei rhodensiense*
			*T. brucei equiperdum*
			*T. brucei evansi*
Stercoraria	*Herpetosoma*	*T. lewisi*	
		*T. rangeli*	
	*Megatrypanum*	*T. melophagium*	
	*Schizotrypanum*	*T. cruzi*	
		*T. dionisii*	*T. dionisii breve*
		*T. vespertilionis*	
	Trypanosomes with unspecified subgenus	*T. evotomys*	
		*T. grayi*	
		*T. grosi*	*T. grosi kosewiense*
		*T. pestenai*	
		*T. theileri*	
		*T. nabiasi*	
	Unclassified trypanosomes	Fish trypanosomes	
		Other unclassified trypanosomes	

**Table 2:** *Trypanosoma* spp. naturally occurring in domestic and wild mammals and in vectors in Europe.

*Trypanosoma* sp.	Country	Host	References
*T. dionisii*	Bulgaria	Bat and bat flies (*Nycteribia shmidlii*)	[73]
	Czech Republic	Bat and bat flies	[73]
	Poland	Bat and bat flies	[73]
	Portugal	Bat	[74]
	United Kingdom	Bat and bat flies	[75]
*T. dionisii breve*	France	Bat	[76]
*T. evotomys*	Bulgaria	*Mus macedonicus*	[77]
	Germany	Voles	[78,79]
	United Kingdom	Voles	[69,80,81,82,83]
	Poland	Voles	[84,85]
*T. grosi*	Czech Republic	*Apodemus agrarius*	[86]
	France	*Apodemus sylvaticus*	[87]
	Germany	Voles	[88]
	Hungary	Voles	[89]
	Ireland	Small rodents	[90]
	United Kingdom	Small rodents	[80,82,90]
	Russia	*Apodemus sylvaticus*	[91,92]
*T. grosi kosewiense*	Poland	Voles	[93]
*T. lewisi*	Finland	*Rattus norvegicus, R. rattus*	[94]
	Norway	*Clethrionomys glareolus, Microtus agrestis; Apodemus sylvaticus*	[95]
	Poland	*Rattus norvegicus*	[96]
	United Kingdom	*Rattus norvegicus*	[82]
	Spain	*Rattus norvegicus, R. rattus*	[41]
*T. melophagium*	Croatia	Sheep ked (*Mallophagus melophagium)*	[97]
	Germany	Sheep ked	[98]
		Sheep	[99,100]
	Holland	Sheep	[101]
	Russia	Sheep	[102]
	United Kingdom	Sheep	[103,104,105,106]
		Sheep ked	[107]
	Former Yugoslavia (Croatia and Kosovo)	Sheep	[108]
*T. musculi*	United Kingdom	Mouse (*Mus musculus*)	[82]
*T. nabiasi*	France	Wild and domestic rabbits	[109]
	Spain	Rabbits (*Oryctolagus cuniculus*)	[110,111]
	United Kingdom	Rabbits	[112]
*T. pestenai*	France	European badger (*Meles meles*)	[113,114]
	Germany	Dog	[115]
	Ireland	Badger	[116]
	United Kingdom	Badger	[117,118,119,120,121]
*T. stefanskii*	Poland	Roe deer (*Capreolus capreolus*)	[122]
*T. theileri*	Belgium	Cattle	[123]
	Germany	Cattle	[124,125]
		Fallow deer (*Dama dama*), red deer (*Cervus elaphus*), roe deer	[126,127]
	Italy	Cattle	[128]
		River buffalo (*Bubalus bubalis*)	[129]
	Ireland	Calf	[130]
	Poland	Cattle	[131]
		European bison (*Bison bonasus*)	[132,133,134,135]
	Sweden	Cattle	[122]
	Spain	Cattle	[136,137]
*T. vespertilionis*	Bulgaria	Bat and bat flies	[73]
	Czech Republic	Bat and bat flies	[73]
	France	Bat	[138]
	Italy	Bat	[139,140,141]
	Ireland	Bat	[142]
	Poland	Bat and bat flies	[73]
	United Kingdom	Bat	[143,144,145]
		Bat and bat flies	[75]
*T. theileri*-like	Italy	Sandflies (*Phlebotomus perfiliewi*)	[146]
	Poland	Deer ked (*Lipoptena fortisetosa* and *L. cervi*)	[147]
	Sweden	Roe deer, fallow deer, European elk, red deer, wild boar (*Sus scrofa*)	[148]
	Russia	Horseflies (*Hybomitra tarandina*, *H. muehlfeldi*, *H. bimaculate*, *Chrysops divaricatus*)	[149]
*Trypanosoma* sp.	Germany	Fallow deer, red deer, roe deer	[150,151]
	Italy	Bat and *Cimex* spp.	[152]
	Hungary	Bat and Cimex spp.	[152]
	Poland	Roe deer, red deer, European elk (*Alces alces*)	[153]
		Roe deer	[154]
	Spain	Bat and *Cimex* spp.	[152]
		Red deer	[155]
		Wild rabbits	[156]
	United Kingdom	Common shrews (*Sorex araneus*)	[157]
		Cattle	[114,158,159]

## Data Availability

No new data were created or analyzed in this study. Data sharing is not applicable to this article.
